# Influence of Pressing Schedule and Adhesive Content on the Rheological Behavior of Wood Fiber-Furnish Mats

**DOI:** 10.3390/ma15041413

**Published:** 2022-02-14

**Authors:** Ali Shalbafan, Heiko Thoemen

**Affiliations:** 1 Department of Wood and Paper Science and Technology, Faculty of Natural Resources and Marine Sciences, Tarbiat Modares University, Noor P.O. Box 46414-356, Iran; 2 Institute of Materials and Wood Technology, School of Architecture, Wood and Civil Engineering, Bern University of Applied Sciences, CH-2500 Biel, Switzerland

**Keywords:** medium-density fiberboard, density profile, adhesive content, mat-furnish, modelling, rheology, pressing schedule

## Abstract

In this study, for a better understanding of the hot-pressing process, the influence of adhesive content (AC) on various features of a typical pressing schedule for medium-density fiberboard (MDF) production, including fiber mat compressibility, heat transfer during hot-pressing, density profile and board properties, were evaluated. It was found that increasing the AC (urea formaldehyde) leads to faster heat transfer towards the mat’s central plane, mainly due to higher heat release from the adhesive polycondensation reaction. Moreover, the results indicate that the time needed to reach the critical mark of 100 °C in the central plane of the mat depends on the duration of the first densification level (FD). Importantly, the pressure peaks (p_max_ and p_2nd_) needed for mat densification are significantly reduced when increasing the AC, which might be attributed to the slippery effect created by the adhesive on the fiber surfaces. The duration of the FD also showed obvious effects on the intermediate density maxima (ρ_inter_) and the core layer density (ρ_core_). In general, the physical and mechanical properties of MDF panels are significantly impacted by the pressing schedule and AC. All in all, the results of this study are valuable information for refining existing rheological models to improve their accuracy and their ability to simulate the vertical density profile during industrial production.

## 1. Introduction

Demand for wood-based panels (WBPs), as a furniture and building material has increased considerably in recent decades. According to FAO statistics, production of WBPs has increased by an impressive rate of about 10 million m^3^ per year on a worldwide basis since 2000, currently reaching around 368 million m^3^ [1]. In addition, the concept of self-optimizing plants has been introduced as an important control principal for today’s WBP industry, in line with the general trend towards agile and more networked production processes, also labeled as Industry 4.0 [2].

In the WBP industry, although the raw material features (e.g., type of raw materials and their geometry) influence the board properties, the hot-pressing operation is one of the most critical steps of the whole panel production, as the properties of the final product depend heavily on the pressing conditions of the wood-furnish mat [3,4,5]. Further, the hot-pressing process is the most expensive part of the entire production and is, therefore, typically defined as its bottleneck. Consequently, a fundamental understanding of the pressing process is crucial for optimizing production speed, costs and energy consumption, as well as manipulating board properties and for the development of new technologies and products [6,7].

In the press, the loosely formed mat of resinated wood fibers or particles is consolidated using high temperature and pressure. The rheological properties of the material depend interactively on temperature, moisture content, adhesive type and its composition and content [8]. Hence, the non-uniform temperature and moisture distribution that prevails during the earlier stages of the pressing cycle causes a differential densification of the mat, establishing a cross-sectional density profile. When aiming to optimize the properties of WBPs, the vertical density profile provides valuable information about the production process and board characteristics. Therefore, manipulation of the vertical density profile is an important key to guarantee both efficiency and productivity of production lines [9]. The density profile dramatically influences most properties of the final product. In general, the bending properties are mostly influenced by the surface layers’ density, and the internal bond strength is affected by the core layer density [10].

Although considerable efforts have already been made to better understand the formation of the vertical density profile, the determination of simple links between single production variables and output parameters is difficult for a process in which many interacting mechanisms affect each other in a rather complex way [11]. To develop a scientifically based method to quantify the impact of variations in pressing conditions on both process and final product, several decades ago researchers proposed an integrated approach that considers those variables important during hot pressing simultaneously, rather than in isolation [9,12]. With this approach, along with increasing computational power, the foundation was laid to develop process models based on fundamental principles. Some analytical models of the hot-pressing process have been presented during the last decades [3,13,14,15,16,17]. Based on these works, analytical models are now used for online prediction of the density profile in continuously operating, wood-based panel presses.

In order to apply such analytical models, the instantaneous and time-dependent compaction behavior of the wood-based mat must be known. To characterize the mat behavior, measurements were already carried out more than 20 years ago (e.g., [17,18]). In these tests, some typical steps of industrial pressing programs, e.g., compaction or unloading steps, were investigated in isolation, and the response of the mat to these actions was measured. However, tests performed with realistic pressing programs show that putting together the results of individual steps is not sufficient to fully describe actual mat behavior. Therefore, further aspects of pressing programs commonly used in industry, which had not previously been explicitly investigated, are described here.

Literature review showed that little work has been published so far to consider the adhesive and its effects on the material’s compressive properties in relation to the pressing schedules. Bolton et al. [19], later confirmed by Pichelin et al. [20], described that the bonding strength of the adhesive is the predominant factor influencing stress relaxation in the mat. Heinemann [21] proposed various concepts to model adhesive cure and its effect on the development of the mechanical board properties during hot-pressing. Clearly, the influence of the adhesive on the rheological behavior of the wood-furnish mats must be considered if a comprehensive understanding of the processes during hot pressing is to be achieved.

Accordingly, the main objective of the work presented here is to determine the effects of selected features of a typical pressing schedule on mat response, cross-sectional density profile and on the resulting mechanical properties of medium-density fiberboard (MDF). These features include the duration of first densification level (FD), the level of degassing step after the main densification (DL), as well as variations in the adhesive content (AC). Such information is needed to further improve our understanding of the hot-pressing process and for refining existing rheological models to improve their accuracy and their ability to simulate the vertical density profile of MDF.

## 2. Experimental Procedure

### 2.1. Materials

Industrial thermo-mechanical pulp fibers for MDF production, mainly from pine and spruce, with moisture content of 5.9% were used for producing the laboratory MDF panels. Urea formaldehyde adhesive (UF) for fiber resination was supplied from BASF (Kaurit 345, Ludwigshafen, Germany) having a solid content of 66%, pH of 8.6 and density of 1.3 g/cm^3^. Ammonium sulphate as hardener (1% based on UF solid content) supplied from Sigma-Aldrich Chemie GmbH (Merck, Buchs, Switzerland) was added to the resin prior to spraying.

### 2.2. Panel Manufacturing

The adhesive mixture was sprayed onto the fiber furnish tumbling in a laboratory rotating drum-type blender via a compressed air spray head. The speed and time for resination was kept constant at 88 rpm and 6 min, respectively. After blending, the fiber mat was manually formed by hand into a 600 mm × 400 mm forming box. Afterwards, the whole mat was prepressed and put into the computer-controlled, lab-scale, single-opening hot press (Hoefer Presstechnik GmbH, Taiskirchen, Austria). The press was operated in the position-control mode; no stops or similar devices were used. Pressing temperature and time were kept constant for all panels at 180 °C and 240 s, respectively. Core-layer temperature and specific pressure were recorded for all panels during hot-pressing.

**Figure 1:**
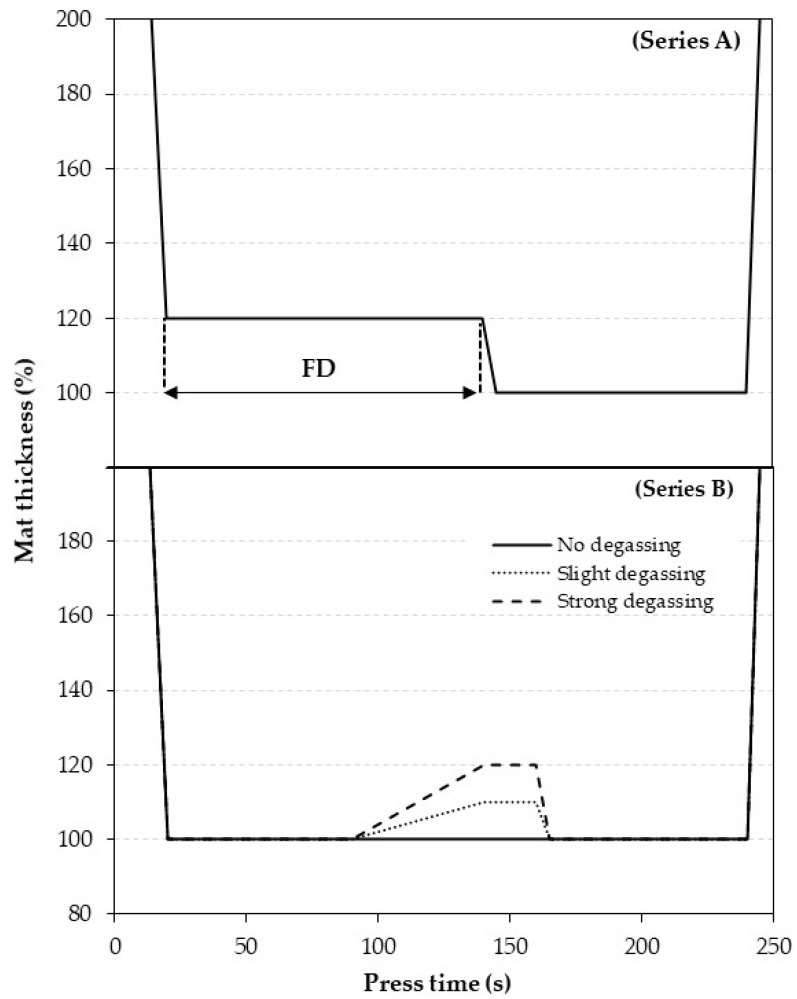
Pressing schedule for MDF manufacturing. (**Series A**) Pressing schedule with different duration of first densification level (FD) (schedule encoded A, B, C and D in Table 1); (**Series B**) pressing schedule for mat degassing behavior evaluation (schedule encoded E, F and G in Table 1). Slight and strong degassing dominate to 10 and 20% level of degassing based on final mat thickness, respectively.

MDF panels were produced with two different pressing series. In series A, the panels were produced with different adhesive content (0, 5 or 10% based on oven dry mass of wood fiber) to evaluate the effect of the duration of the first densification level on core layer temperature and specific pressure as well as on the physical and mechanical properties of the panels. In series B, the influence of the intermediate degassing level (10 or 20% higher than final mat thickness) was investigated only on the temperature and specific pressure of the mat. Regardless of adhesive content, the mat moisture content (including the moisture from the adhesive) was kept constant in all panels at 10%. More details about the pressing schedule for the panel production and mat composition are presented in Table 1 and Figure 1. Except panel A, the final target density and thickness of panels were kept constant at 710 kg/m^3^ and 16 mm, respectively.

Please note that the 100% mat thickness in Figure 1 is equal to the final panel thickness (16 mm). A duration of first densification equal to 0 s means that the panel thickness never reached its 100% thickness level (code A with final thickness of 19.2 mm).

Mats E, F and G were prepared without adhesive, but still had a constant mat moisture content of 10% to evaluate the effects of mat degassing behavior during hot-pressing on the mat’s dependent variables (core layer temperature and specific pressure). Mat E is considered as a reference pressing schedule with neither opening nor densification during pressing. Mats F and G are considered as strong and slight degassing pressing schedules, respectively. For each pressing schedule, three panels were manufactured, resulting in a total of 45 panels.

### 2.3. Data Acquisition

The effects of the duration of first densification level on the panel’s density profile were measured. To this end, the vertical density profile was measured using a laboratory X-ray device (DAX 5000, Fagus-GreCon GmbH, Alfeld, Germany). Scanning speed was set to 1 mm/s with a resolution of 0.02 mm. Three 50 mm × 50 mm samples were cut from the middle of each panel and were conditioned at 20 °C and 65% relative humidity prior to measurement.

The thickness of each sample was normalized to 100% to cope with slight variations in the samples’ thickness due to unequal springback effects. The presented density profiles are averaged over nine samples in each code number. To describe each density profile, values for the following characteristic were determined:

- Density maxima near the sample surfaces (ρ_surf_) averaged over the top and bottom of the panel.
- Intermediate density maxima (ρ_inter_). This value is only presented for panels B, C and D.
- Average core density (ρ_core_) between 47.5 and 52.5% of the panel thickness.
- Ratio of ρ_inter_/ρ_core_.

Two characteristic values were also derived from the recorded core layer temperature (named t_100°C_) from the beginning of the pressing process until 100 °C was reached in the central plane, and T_240 s_ denominating the core temperature just before press opening. Moreover, three characteristic values were derived from the specific pressure curves entitled p_max,_ p_2nd_ and p_240s,_ which correspond to the maximum pressure during first densification, maximum pressure during second densification step and the pressure at the end of the pressing cycle, respectively. Please note that p_2nd_ is not presented in panels A and E due to their having a constant mat thickness level during hot-pressing.

### 2.4. Panels’ Properties

All samples were conditioned at 65% relative humidity and 20°C until a constant mass was reached. The mechanical properties of the MDF were analyzed by three-point bending test (bending strength and bending modulus) and internal bond strength according to EN310 and EN319, respectively [22,23]. All mechanical tests were performed using a Zwick/Roell apparatus (Ulm, Germany). Thickness swelling (TS) and water absorption (WA) of the MDF were measured after 2 and 24 h of water soaking according to EN 317 [24]. Three replicates of each panel (n = 9) were randomly selected for testing.

One-way analysis of variance was conducted using SPSS software program version 26 (IBM Corp., Armonk, NY, USA) for statistical data analysis.

## 3. Results and Discussion

### 3.1. Mat Temperature

**Figure 2:**
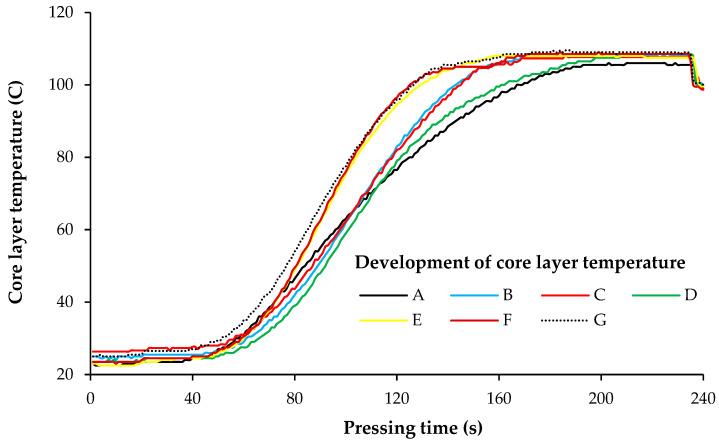
Temperature development for different pressing schedules, measured in fiber mats without adhesive.

The typical temperature curves for different pressing schedules, measured in wood fiber mats without adhesive, are illustrated in Figure 2. The core layer temperature started to increase after nearly 50 s of pressing time in all panels, then rapidly reached 100 °C and maximum temperature, and eventually was constant prior to press opening. Approximately similar graphs were observed in fiber mats with different AC. The characteristic values of the temperature curves are also shown in Table 2. The time to reach 100 °C in the central plane of the mat (t_100°C_) is strongly influenced by the FD. The shorter the first densification level, the shorter t_100°C_. In other words, faster compaction of the fiber mat towards the thickness level of 100% (equal to final panel thickness) leads to a faster increase of temperature in the central plane. Moreover, the mat thickness level shows a strong influence on the time t_100°C_. The 20% higher level of code A compared to code E leads to a nearly 40 s delay in reaching 100 °C. Clearly, the lower density of the mat following code A results in reduced thermal conductivity and, hence, in reduced heat transfer. This finding is in accordance with that presented by Thoemen and Ruf [11].

The degassing step (codes F and G compared to code E) around halfway through the pressing cycle slightly accelerated the time to reach 100 °C. Overall, the lowest values for t_100°C_ were observed in panels with a degassing step. The maximum temperature (T_240s_) just before press opening averaged around 106–108 °C shows a statistically significant correlation with the time to reach 100 °C. As expected, a faster increase of the temperature in the central plane leads to a slightly higher final temperature in this layer.

Table 2 also shows that the time to reach 100 °C in the mat’s central plane is influenced by the adhesive content. The temperature reached 100 °C faster with increasing adhesive content in all pressing schedules. This can be attributed to the heat released from the exothermal polycondensation reaction of the UF adhesive during hot curing, which accelerates the temperature evolution inside the mat [21,25].

### 3.2. Specific Pressure

**Figure 3:**
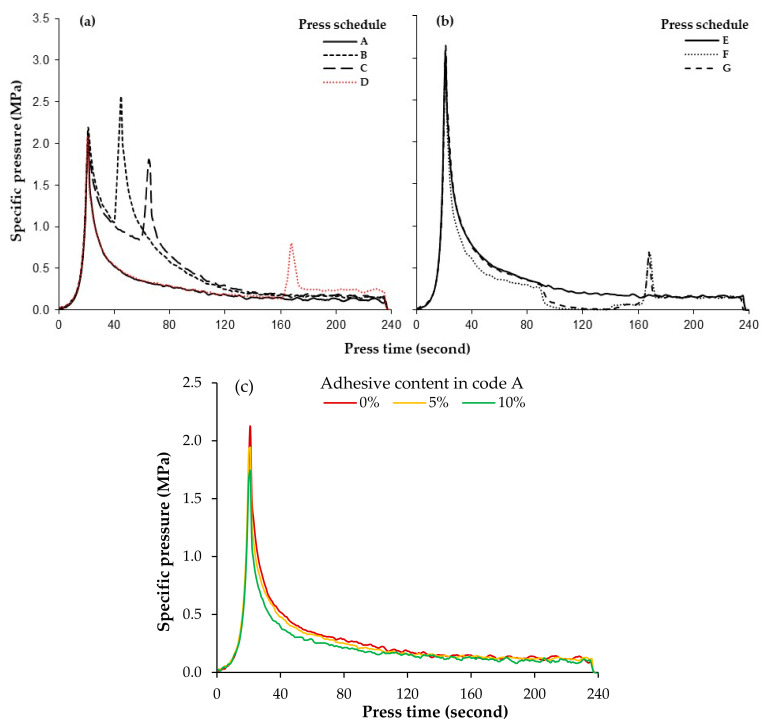
Specific pressure for different pressing schedules, measured on fiber mats without adhesive: (**a**) pressing schedules with different duration of first densification level; (**b**) pressing schedules with different degassing levels; (**c**) specific press pressure in panels A with different adhesive content.

Typical specific pressure curves for different pressing schedules applied to wood fiber mats without adhesive are illustrated in Figure 3. The maximum pressure (p_max_) is observed early in the pressing cycle, the pressure then rapidly declines, then the secondary peak pressure (p_2nd_) is formed during the final densification step. Eventually the pressure drops to p_240s_ prior to press opening. Approximately similar graphs can be observed for mats with different adhesive contents.

Characteristic values for the specific pressure are also presented in Table 2. As expected, the duration of first densification (code A, B, C and D) has no influence on the maximum pressure (p_max_). However, p_2nd_ strongly depends on the duration of first densification. The shorter the duration of first densification, the higher p_2nd_. The highest value for p_2nd_ was observed in panels with the shortest first densification level (20 s in panel B), having an even 18% higher pressure value compared to its corresponding p_max_. Extending the first densification level up to 140 s significantly reduces p_2nd_ to a value considerably lower than p_max_ and slightly raises p_240s_. In contrast, there is no significant effect of the degassing step on any of those characteristic values of the specific pressure (p_max_, p_2nd_, p_240s_). These findings are consistent with those observed by Thoemen and Ruf [11].

The pressure p_max_ is more than 3 MPa in panels reaching final panel thickness immediately after press closing (see Figure 3b). In other words, the mat thickness level after first densification shows a clear influence on all specific pressure values. The more the mat is compressed early in the pressing cycle (codes E to G compared to A to D), the higher the maximum pressure (p_max_) applied for mat densification.

Another important finding from the data presented in Table 2 is the influence of adhesive content on all specific pressure values. As shown in Figure 3c, the maximum pressure (p_max_) needed for mat densification is significantly reduced by increasing the adhesive content up to 10%. Depending on the pressing schedule, a reduction in p_max_ of about 10–18% is observed with increasing AC. A similar trend can be seen for p_2nd_ when increasing the AC: the higher the AC, the lower the specific pressure needed for mat densification. This may be attributed to the slippery effect created by the adhesive on the fiber surfaces. Adhesive acts like a soap on the fiber surfaces, improving the compressibility of the fiber mat. It can also be seen that the pressure drops faster after reaching the target thickness when using a higher content of adhesive, showing a faster stress relaxation compared to mats pressed without adhesive [19,20]. The results also show that the AC has no effect on p_240s_. The adhesive is fully cured at the end of the pressing process, and a stiff web of fibers is created.

### 3.3. Density Profile

The effects of the pressing schedule on the overall density maxima near the panel surfaces (ρ_surf_), intermediate density maxima (ρ_inter_), core density (ρ_core_) and the ratio of ρ_inter/_ρ_core_ are illustrated in Table 3. No pronounced impact of the duration of first densification on the maximum density ρ_surf_ was observed. This finding confirms that actions far into the pressing cycle do not influence the maximum surface layer density [11,16,26]. The maximum density (ρ_surf_) is mainly affected by the maximum pressure (p_max_). As seen in Table 2, p_max_ is similar in all panels manufactured with different durations of FD, resulting in similar ρ_surf_.

Intermediate density maxima (ρ_inter_) between surface and central plane occur most clearly for panels B, C and D. The duration of first densification shows the most marked effect on ρ_inter_. For a better comparison, the ratio between ρ_inter_ and the core density (ρ_core_) is used to analyze the height of the intermediate density maxima. For example, a ratio of 1.2 denotes that the intermediate maximum density exceeds the average core density by 20%. The highest ratio of ρ_inter_/ρ_core_, around 1.6, was observed in those panels with shortest duration of first densification (code B). This ratio of ρ_inter_/ρ_core_ significantly declines with increasing duration of first densification to 140 s (code D).

**Figure 4:**
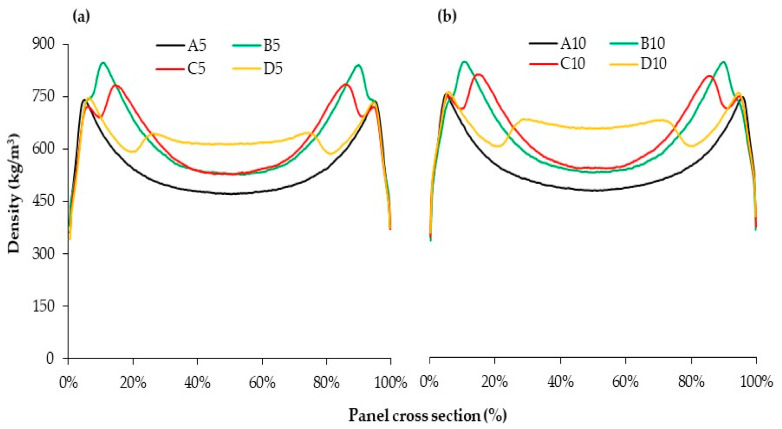
Density profile curves for different pressing schedules of fiberboard with different adhesive contents: (**a**) panels with 5% adhesive content; (**b**) panels with 10% adhesive content.

The duration of first densification also has an obvious effect on the position of the intermediate density maxima between surface and central plane of the panel. The position of the intermediate density maximum moves closer to the panel center with increasing duration of first densification (Figure 4).

Table 3 also shows that the core layer density (ρ_core_) is influenced by the duration of first densification. The lowest core density was observed in panels with no densification step (code A) after press closing where the mat thickness level was kept constant at 120%.

The two early densification steps, after 20 and 40 s in panels B and C, respectively, had a similar impact on the core layer density. A longer first densification step (140 s in panel D) increases the core layer density by about 20% compared to those with shorter durations of first densification. This is because of the core layer temperature, which is close to room temperature when densified after 20 or 40 s, but has reached almost 100 °C when densified after 140 s (see Figure 2). The higher the core layer temperature, the higher the compressibility of the wood fibers and, accordingly, the higher the core layer density [8,12,14].

Figure 4 shows various density profiles of panels pressed with different pressing schedules and AC. A highly symmetric density profile for each panel can be observed. A closer examination reveals that the adhesive content has no noticeable impact on the characteristic values of density profiles. Figure 4 also suggests that the overall density minimum switches from the core to an intermediate position when the second densification is delayed (code D), resulting in a relatively high core density.

### 3.4. Bending Properties

**Figure 5:**
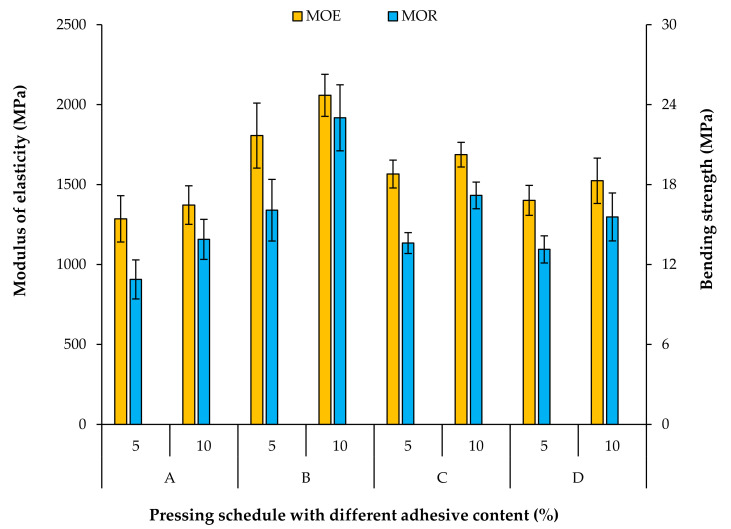
Bending properties of fiberboards produced with different pressing schedules and adhesive contents.

The effects of various pressing schedules and adhesive contents on the bending properties of the fiberboards are illustrated in Figure 5. Both factors show a significant impact on bending strength (MOR) and modulus of elasticity (MOE). Although the density maxima near the surfaces (ρ_surf_) in all panels are nearly similar, the highest MOR and MOE is observed in panels produced with the shortest first densification level (code B). This does not surprise, as an early densification to the final mat thickness level of 100% leads to intermediate density maxima with ρ_inter_ surpassing ρ_surf_. Further, the intermediate density maxima are much closer to the surface layers, which positively influences the bending properties. The highest ρ_inter_ was achieved in panels with lowest duration of first densification (code B), followed by panels C and D; the same order is visible for the bending properties. The lowest MOR and MOE is achieved in panels without a second densification step (code A) where the mat thickness level was kept constant at 120% during the whole pressing cycle. This result does not surprise, as the higher mat thickness level leads to a lower final panel density. The panel density is a significant factor that influences most panel performance indicators [27].

Figure 5 also shows that both MOR and MOE values have positive correlations with the adhesive content. In all pressing schedules, both MOR and MOE rise with increasing adhesive content up to 10%. This increasing effect in bending properties is more pronounced in panels with shorter first densification steps. This finding is also compatible with the results reported by other researchers [3,10,16]. A higher content of adhesive creates a stronger link between the fibers and increases the bending resistance of the panels [28,29].

**Figure 6:**
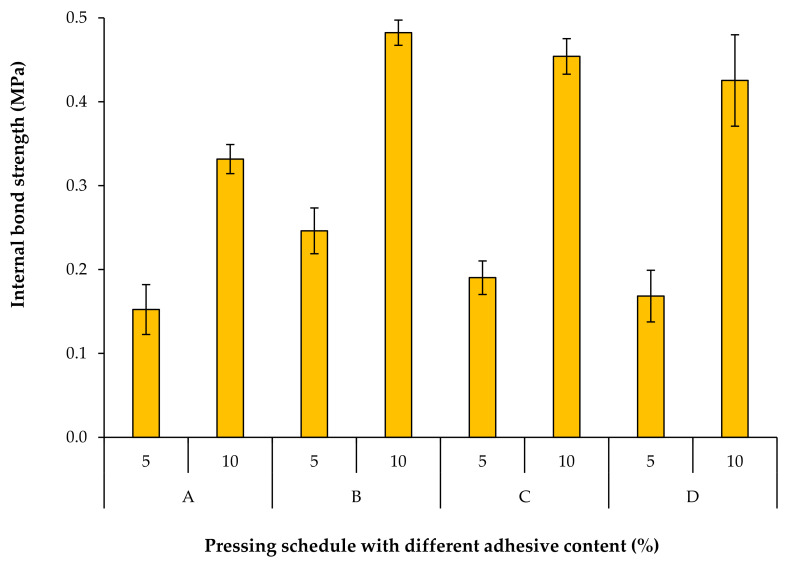
Internal bond of fiberboards produced with different pressing schedules and adhesive contents.

Further analysis was accomplished to evaluate the effects of different pressing schedules and AC on the internal bond strength (IB) of the fiberboards (Figure 6). The lowest IB value was achieved in panels without a second densification step (code A) where the mat thickness level was kept constant at 120% till the end of the pressing schedule. The core density was also the lowest in these panels, causing the lower IB value. Results also show that the IB value decreases when increasing the duration of first densification, although such difference is more pronounced in panels with a lower AC. Although the core layer density is highest in panels with the longest first densification step (code D with 140 s), the IB value is still lower compared to those with a shorter first densification step (code B and C). This can be attributed to two main reasons. A closer look at Figure 4 shows that the lowest density in panel D is in the transition zone from the surfaces towards the core layer (intermediate position) close to the intermediate density maxima. Presumably, the failure in the IB tests happens at this position and results in lower IB values. Moreover, the delayed second densification step can, to some extent, deteriorate the already cured glue bonds in intermediate or inner layers, which may negatively influence the IB value. Data presented in Table 2 also show that the temperature required for adhesive curing is reached in the core layer prior to the second densification step.

Figure 6 also displays that the IB values are positively correlated to adhesive content. Indeed, the IB strength is related to the evolution of the adhesive bond strength development within the board. The IB was more than doubled by increasing the adhesive content from 5% to 10%. This finding is compatible with the results reported by previous researchers [21,25,29,30]. Better connections between the wood fibers can be established by increasing the adhesive content.

**Figure 7:**
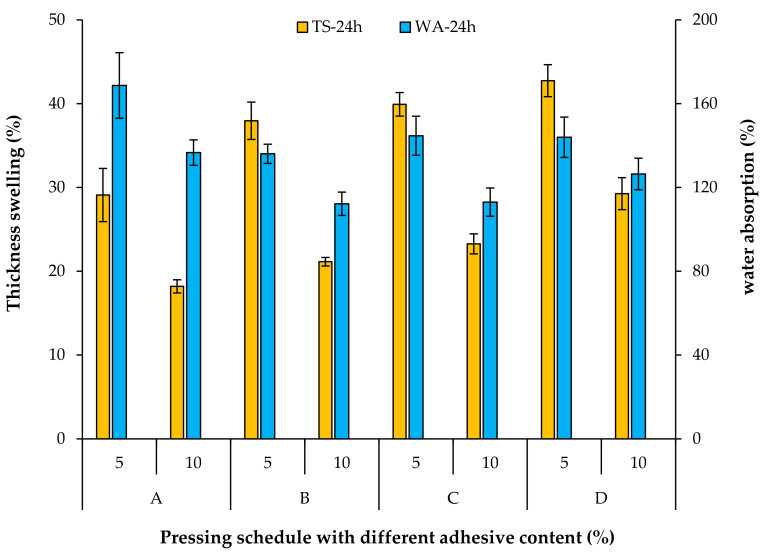
Thickness swelling and water absorption of fiberboards produced with different pressing schedules and adhesive content.

Thickness swelling (TS) and water absorption (WA) are properties to determine the dimensional stability and water soaking of fiberboards. Figure 7 shows that TS and WA values after 24 h water soaking are impacted by the pressing schedule. The lowest TS-24 h was observed in panels without a second densification step (code A). It is believed that the TS value in wood-based panels results from the sum of two major effects; swelling of the wood material and springback of the panel after pressing, due to released compression stresses from pressing operation [31,32]. Please note that panel A was less compressed compared to those panels having a second densification step, and less swelling can be anticipated for it during water soaking. The TS-24 h values also raise when increasing the duration of first densification from 20 s to 140 s. This is likely due to the weaker interaction between the wood fibers, which is also reflected by the reduced internal bond values [32].

The highest WA-24 h was observed in panel A produced without a second densification step. More voids are available between the fibers of panel A due to less compaction, which makes it easier for water to penetrate the panels. A trend similar to that of TS was also observed for WA in panels with various durations of first densification. The higher the duration of first densification, the higher the WA.

It is also visible from Figure 7 that both TS and WA values reduce significantly with increasing of AC. TS and WA values improve nearly 40% and 20%, respectively, with an increase in the AC by 5 percentage points. Apparently a higher AC creates more bonds between wood fibers, which prevents water absorption and improves the dimensional stability of the fiberboard.

## 4. Conclusions

The temperature reached 100 °C faster in the mat central plane due to heat released from UF polycondensation reaction when the content of UF adhesive was increased. Moreover, the mat thickness level showed more pronounced influence on the time to t_100°C_ than that of first densification level. The 20% higher mat thickness level led to nearly 40 s delay in reaching t_100°C_. Adhesive showed a soapy effect on fiber surfaces, which increased the fiber compressibility. The higher the AC, the lower the specific pressure needs for mat densification, which would be an important criterion during hot-pressing control. The duration of FD affects the density profile in three ways. First, when the duration of FD is increased, the core layer density raised about 20% compared to those with shorter densification time; second, the position of the ρ_inter_ shifts toward the central plane of the mat; third, the highest ratio of ρ_inter_/ρ_core_, around 1.6, was observed in panels with shortest first densification time. It also appears to be reasonable to conclude that there is only a minor impact of AC on the density profile development. The highest ρ_inter,_ which positively influenced the bending properties, was achieved in panels with lowest duration of FD. In all pressing schedules, bending properties were raised with increasing AC up to 10%, with the trend more pronounced in panels with shorter duration of FD. Extension of first densification step towards press ending, which led to a delayed second densification step, negatively influenced the IB, TS and WA values mainly due to the deterioration of the already cured glue bonds in intermediate or inner layers.

The results presented in this paper do not only contribute to better understand the behavior of the wood-furnish mat during hot-pressing of WBPs, they also provide valuable information for further developing computer-based simulation models of this process. Particularly, the time-dependent behavior of the mat and the influence of the adhesive on the mat’s response are important mechanisms which so far are incompletely understood. Computer-aided models for the simulation of the WBP process will soon arrive in practice and represent powerful tools for optimizing the industrial WBP process from a technological, economic and ecological point of view.

## Figures and Tables

**Table 1:** Protocols used for panels production.

	Pressing Schedule						
Independent Variables	Series A				Series B		
Duration of 1st densification level (s)	−	20	40	140	−	−	−
Final mat thickness (%)	120	100	100	100	100	100	100
Degassing level (%)	0	0	0	0	0	10	20
Sample code	A	B	C	D	E	F	G
**Adhesive content (%)**							
0	*✓*	*✓*	*✓*	*✓*	*✓*	*✓*	*✓*
5	*✓*	*✓*	*✓*	*✓*	*	*	*
10	*✓*	*✓*	*✓*	*✓*	*	*	*

Minus (−) means the first densification is not relevant for this panel. Tick (*✓*) means panels produced with this protocol. Asterisk (*) means no panels prepared with this protocol. Mat thickness level of 100% means final panel thickness of 16 mm.

**Table 2:** Characteristic values of temperature and specific pressure curves for various pressing schedules.

Pressing Schedule	Adhesive Content (%)	Temperature		Pressure		
		t_100°C_	T_240 s_	p_max_	p_2nd_	p_240s_
		Second	°C	MPa	MPa	MPa
A	0	167	106	2.12	-	0.10
B		142	108	2.17	2.56	0.13
C		145	108	2.02	1.72	0.15
D		159	108	2.08	0.80	0.22
E		129	108	3.03	-	0.17
F		125	109	3.06	0.66	0.15
G		125	109	3.15	0.69	0.15
A	5	150	106	1.94	-	0.12
B		128	108	2.05	2.44	0.12
C		141	108	1.80	1.58	0.13
D		151	108	1.83	0.75	0.20
A	10	142	106	1.74	-	0.10
B		120	108	1.94	2.22	0.12
C		132	108	1.65	1.41	0.13
D		146	108	1.74	0.69	0.20

**Table 3:** Characteristic values of density profile for various pressing schedule.

Pressing Schedule	Adhesive Content	Maximum Surface Density (ρ_surf_)	Maximum Intermediate Density (ρ_inter_)	Core Density (ρ_core_)	Ratio of ρ_inter_/ρ_core_
A	5	743	-	470	-
	10	727	-	462	-
B	5	740	857	530	1.62
	10	725	820	513	1.60
C	5	722	785	527	1.49
	10	724	782	525	1.49
D	5	737	644	614	1.05
	10	739	663	632	1.05

## Data Availability

The data presented in this study are available on request from the corresponding authors.
